# Care-giving as a Canadian-Vietnamese tradition: ‘It's like eating, you just do it’

**DOI:** 10.1111/hsc.12126

**Published:** 2014-10-20

**Authors:** Rhonda Donovan, Allison M Williams

**Affiliations:** 1 Wilfred Laurier University-BrantfordBrantford, Ontario, Canada; 2 School of Geography and Earth Sciences, McMaster UniversityHamilton, Ontario, Canada

**Keywords:** culture, end-of-life family caregivers, health geography, qualitative research, Vietnamese

## Abstract

The objective of this study was to examine how Vietnamese family caregivers (FCGs) perceive, manage and experience end-of-life care-giving for seriously ill family members. Using an instrumental case study design, this longitudinal qualitative research employed the use of cultural brokers/language interpreters to help ensure that the research was conducted in a culturally-appropriate manner. Participants (*n* = 18) discussed their experiences of care-giving within the context of a traditional cultural framework, which was found to influence their motivations and approaches to care-giving, as well as their propensities towards the use of various supports and services. The study was carried out in southern Ontario, Canada, and participants were providing home-based care-giving in the community. Data were collected throughout 2010 and 2011. The ways in which care-giving was perceived and expressed are reflected in three themes: (i) Natural: identity and care work; (ii) Intentional: whole-person care; and (iii) Intensive: standards, struggle and the context of care. This research confirms the need for culturally-appropriate services and supports while illustrating that Vietnamese FCGs not only value, but are also likely to use healthcare and social services if they are language-accessible, built on trust and demonstrate respect for their values as individuals, regardless of culture.

**What is known about this topic**

- Cultural identity is an important factor which impacts both perceptions and responsibilities for care, including the capacity to adapt and cope with care-giving, illness, dying, death and bereavement.

**What this paper adds**

- This paper presents the first ‘world’ view of the experience of family care-giving from the perspectives of Vietnamese family caregivers living in Canada.
- Findings confirm the need for culturally-appropriate health and social services and supports, characterised as: language-accessible; family-oriented (rather than patient-centred); respectful of privacy and modesty; and tolerant of multiple healthcare approaches (i.e. both western and traditional medicine), as well as specific food preferences.

## Introduction

### Family care-giving in Canada

In Canada, enhancing access to palliative and end-of-life (P/EOL) care is a major policy and practice issue. This includes improving support for family members and friends, who have become, within the context of healthcare restructuring, informal caregivers to their aged, seriously ill and dying kin in the home. While the demands of care-giving can adversely affect the health of family caregivers (FCGs), access to supportive services that can mitigate burden is often inadequate. For example, specialised P/EOL care services – typically provided under the rubric of home care – are accessible to about only 30% of the population (Carstairs 2010). The development of services for FCGs is impeded by the lack of understanding about their specific needs. Most research into family care-giving at end of life in Canada has focused on the perspectives of FCGs within the context of various disease categories of the care recipient or across different care settings, rather than in relation to FCGs’ specific ethnic or cultural identities. Such homogeneity belies the impact of culture, among other social and demographic factors, on the experiences and outcomes of P/EOL care-giving and relationships with home place. Given Canada's growing multicultural population, this knowledge is critical to inform the development of appropriate supports and services which can minimise negative impacts and enhance the care-giving experience. The purpose of this case study was to examine the experiences of Vietnamese-Canadian FCGs who are providing care in the home to understand how best to support them, the care recipient and their families.

Given the success of the population health promotion (PHP) model in framing research into care-giving, it is employed herein as a conceptual framework (Donovan *et al.* 2010). As detailed elsewhere (Donovan *et al.* 2010), the PHP model is a framework that guides actions to improve health (Hamilton & Bhatti 1996) through advocating for simultaneous consideration of the determinants of health, the ways by which inequities can be addressed and the scalar dimensions that should be targeted to achieve this.

As is well documented (Funk *et al.* 2010, Stajduhar *et al.* 2010), numerous challenges are faced by FCGs when caring for dying kin in the home, many of which can be mediated by access to healthcare services. Accessing services and supports is problematic for FCGs in general (Guberman & Maheu 2003/2004). This is exacerbated for minority populations, such immigrant groups, which do, despite universal healthcare in Canada, face problems accessing services for care and support, both in general and specific to P/EOL care. This includes barriers to services due to geographical (Access Alliance Multicultural Community Health Centre 2005), linguistic, cultural or religious differences and different health beliefs (Krakauer *et al.* 2002, Hotson *et al.* 2004, Siriwardena & Clark 2004, Szczepura 2005). Immigrant populations experience unmet healthcare needs, are unsure as to where to access services and believe that care will be inadequate (Wu *et al.* 2005). Furthermore, the need to access culturally-appropriate healthcare may be heightened at end of life as people are more likely to draw upon various religious and cultural beliefs, practices and rituals to cope with the fear, stress and grief associated with dying (Dilworth-Anderson *et al.* 2002, Krakauer *et al.* 2002, p. 184). Many ethnic minorities in Canada and elsewhere share a commitment to the role of providing care in the home (Somerville 2001, Mok *et al.* 2003). As suggested by Turner *et al.* (2004), there is a wide range of factors influencing the healthcare decisions families make, including trust in the healthcare system and in its care providers – all of which vary cross-culturally.

### A case for the Vietnamese

With a population of just over 180,000, the Vietnamese are the fifth largest visible minority population in Canada. Approximately 64% (115,000) Vietnamese–Canadians are immigrants (Statistics Canada 2007). The largest proportion of immigrants (73,850) arrived in Canada prior to 1991; this was one of four separate waves (Richard & Dorais 2003), two of which were dominated by refugees. The implications of the conditions of migration on their physical and psychological health, and material, social and economic well-being in space and time are tremendous, indelibly engrained in their psyche. In terms of language, most report a non-official language (neither English nor French) as their mother tongue and the language most spoken at home. However, 88% of Vietnamese-Canadians can speak either English or French, while 12% cannot speak either (Statistics Canada 2007).

Early research and reports concerning healthcare encounters with Vietnamese families emanate largely from the United States (Purnell 2008). However, much of this research is outdated, and has focused primarily on specific healthcare practices and diseases (Calhoun 1985, Die 1988, Jenkins *et al.* 1996, Free *et al.* 1999, Purnell 2008) rather than family care-giving and palliative/end-of-life (P/EOL) care specifically (Strumpf *et al.* 2001, Tran *et al.* 2006, Liu *et al.* 2008). This work tells us that, similar to other Asian cultures, the Vietnamese favour a collectivist orientation towards family care; show a propensity towards privacy and independence; and often combine biomedical and traditional healing practices (Purnell 2008). This body of knowledge continues to inform the literatures specific to the delivery of culturally-competent healthcare for Vietnamese populations (see: http://www.stanford.edu/group/ethnoger/); however, it may not necessarily reflect current practices, values and beliefs – especially for Vietnamese-Canadians in particular. No research could be found concerning Vietnamese family care-giving in Canada.

## Research framework and design

Similar to our earlier work, we have operationalised diversity through the use of the term ‘culture’ (Donovan *et al.* 2010). Culture is a macro-level concept that encompasses several components, including ethnicity, religion, gender, socioeconomic status, sexual orientation, health/disability status and geographical region (Guberman & Maheu 2003/2004, Blevins & Papadatou 2006). Culture is not fixed, but fluid and dynamic in space and time; therefore, it does not definitively ‘determine’ behaviour per se; rather, it is known to influence both health-promoting and health-seeking behaviours. In this case, a cultural perspective can help inform understandings of both beliefs and behaviours associated with illness, care-giving, dying, death and bereavement. As the purpose of this study was to gain insight and understanding into the care-giving experience, an instrumental case study design was employed (Baxter & Jack 2008). The study was longitudinal in nature to capture changes to the care-giving situation over time (Donovan *et al.* 2010). Cultural brokers (CBs) were used to help ensure that the research was conducted in a culturally-appropriate and sensitive manner. For example, CBs help identify culture nuances and sensitise the researchers to other perspectives. Specifically, they helped translate documents, such as brochures, consent forms, reports and interview schedules; recruit and interview participants; and debrief and transcribe interview tapes. Ethical approval for this study was obtained from the McMaster University Research Board.

### Site and sample

This study took place in southern Ontario in collaboration with recruitment through CB and various community-level healthcare and social service organisations. As determined by data saturation, a total of 18 FCGs who were providing care in the home were recruited to the study. The sample included both active (*n* = 11) and bereaved (*n* = 7) FCGs who were involved in home-based care at the time of recruitment, or, in the case of bereaved FCGs, where home-based care was involved prior to the death of the care recipient. All participants were female and self-identified as Vietnamese, Vietnamese-Canadian or Vietnamese-Chinese. As outlined in Table 1, FCGs ranged in age from 16 to 80 years and were providing care to a family member. Care-giving situations varied in length, from 3 months to 5 or more years; all care recipients were end of life in terms of the nature of their illnesses, but were not all imminently dying when the caregiver was recruited to the study. Further details concerning the demographic information and the care-giving situation are provided in Table 1.

**Table 1:** Description of family caregiver (FCG) characteristics (*n* = 18)

Characteristics	*n*
Total sample, female caregivers (providing care to 16 family member care recipients*)	
Care-giving status at the time of study participation	
Actively care-giving	11
Bereaved caregivers	7
FCG age‡	
16–25 years	1
26–35 years	3
36–45 years	2
46–55 years	4
56–65 years	4
66–75 years	2
+75 years	2
Marital status†	
Married	10
Single	4
Separated/divorced	4
Time in caregiver role	
<1 year	4
1–5 years	7
+5 years	7
Education completed	
None	1
Primary	2
Secondary	6
Post-secondary+	9
Household income†	
Undisclosed	2
<$20,000	7
$20,000–$40,000	6
$40,001–$60,000	3
Religion	
None/other	2
Western	8
Non-western	8
Immigration status	
Sponsorship	8
Refugee	10
Time in Canada†	
<5 years	1
6–10 years	5
11–15 years	4
+16 years	8
Relationship with CRG	
Spouse	8
Daughter	9
Daughter-in-law	1

* In two cases, two FCGs were providing care to the same care recipient.

† Status at the time of care-giving.

‡ Age of participants was collected as a key demographic variable which can be cross-checked by readers to determine caregiver relationship and cohort of immigrant to Canada.

### Data collection

Along with the assistance of CBs where requested and appropriate, one to five interviews (*n* = 50) were conducted with each caregiver participant, with each interview lasting anywhere from 1 to 3 hours. The number of interviews held with each caregiver respondent varied due to a range of reasons, including availability of time for interview, interest in continuing with study and changes in care-giving trajectory. In addition to the formal interviews, we conducted a pre-assessment and information-gathering interview, lasting anywhere from a half to a full hour; in addition to reviewing the informed consent process, this ensured that the participants met the study eligibility criteria while also confirming participant's interest. The interview guide was based on a review of the literature, as developed and tested in our earlier study with the group of Dutch Reformed FCGs (Donovan *et al.* 2010), and included questions in the following areas: meaning of home, impact of ethnicity, culture and religion, household adjustments, caregiver supports and patient services, financial adjustments, and household and caregiver demographics. The guide was first vetted with our Vietnamese CBs and several caregiver participants in an attempt to ensure cultural appropriateness and sensitivity with this sample (see Box 1).

**Box 1Sample of interview questions asked**

1. Do you feel that health service personnel are willing to be flexible about accommodating to your family?
2. Do you feel that healthcare providers need to know more about your cultural heritage and/or spiritual beliefs to better assist you through care-giving?
3. Are you involved with any groups within your larger community, whether religious, recreational, social or cultural? If so, which are they? Do any of these groups provide support to you in your care-giving role?

The interviews were conducted in the participants’ language of choice (either English or Vietnamese); interviews conducted in Vietnamese were translated in situ by the CB(s), in the presence of the primary researcher. This enabled us to participate in the interviews, probe responses and process the context within which responses and care-giving occurred, a process in which all FCGs were comfortable. To demonstrate our appreciation, participants received a stipend of $25 (CAD) following each interview. In addition to the caregiver participants, we conducted several (*n* = 7) key informant interviews with individuals who were experienced heads of community and social service organisations interested in informing the wider service landscape for Vietnamese caregivers. Key informants were asked about Vietnamese culture, care-giving norms and religion, and/or health and social service provision more generally.

### Data analysis and interpretation

Data analysis occurred simultaneously with data collection to organise the large amount of data that were collected, to direct ongoing data collection and to identify gaps in the data (Miles & Huberman 1994). All interview tapes were transcribed and translated, either verbatim or contextually, as described earlier; potential identifiers were removed to ensure confidentiality. Key informant data were triangulated with the data set to provide context to the analysis. Initial coding and subsequent category development were achieved using Saldana's (2009) pre-existing framework of values and emotion coding. The process of *values coding* was used to understand participant's values, attitudes and beliefs of his or her perspectives or worldview, as expressed in the form of thoughts, feelings and actions (Saldana 2009). *Emotion coding* was used to:

Label the emotions recalled and/or experienced by the participant, or inferred by the researcher about the participant.

Where an emotion is defined as a:

Feeling and its distinctive thoughts, psychological and biological states, and range of propensities to act.

(Saldana 2009, p. 86)

Both methods are appropriate for use in studies that explore intrapersonal and interpersonal participant experiences. These codes were categorised and ultimately developed into three themes that capture how participants *perceived, managed and experienced* care-giving, as now discussed. To ensure methodological rigour, we used several strategies, including prolonged engagement via a series of longitudinal interviews, member checking, peer debriefing, triangulation, reflective journaling and thick description (Baxter & Eyles 1997).

## Findings

Participants spoke about their experiences of care-giving within the context of a traditional cultural framework; this framework influenced their motivations and approaches to care-giving, as well as their propensities towards the use of various supports and services. FCGs typically contrasted their experiences in Canada to what would be expected and possible in Vietnam, particularly within the context of early socialisation processes that occurred prior to migration. For example, thoughts, opinions and feelings about their experiences with care-giving were often prefaced with statements such as: ‘in Vietnam…’ or ‘Vietnamese people…’. The value of caring for family members who are aged, seriously ill or dying is highly engrained in their identity, such that ‘it's like eating, you just do it’ [KI05; Int 1]. The ways in which care-giving is perceived and expressed as features of their cultural and socio-spatial identities are illustrated in the following three themes, entitled: (i) Natural: identity and care work; (ii) Intentional: whole-person care; and (iii) Intensive: standards, struggle and the context of care.

### Natural: identity and care work

This theme represents FCGs’ perceptions towards expectations and structures of care and care-giving. More than just reflections of one's implicit value system, care and care-giving were expressed as important facets of Vietnamese culture: they were seen as natural, essential and central to their identity. Care-giving was understood as a familial expectation:

[s]he [mother] took care of us, so now it is our turn.

[FCG17; Int 1, age 46]

Care for and by the family, particularly for elderly or ill parents, is valued over the needs of the individual, regardless of the caregiver's age or the length of time spent in care-giving. As expressed by one FCG who quit work and moved in with her seriously ill mother:

I'd finish my duty [of caring for my mother] with all my heart; I'll figure it out what I can do for my life later.

[FCG16; Int 2, age 54]

It is critical to note that the expectation for ‘family care’ referred almost exclusively to parental care within the immediate family. For example, adult-children expressed understanding of their *duty* to care for their aged and/or ill parents, but not their married siblings, aunts, uncles or cousins. FCGs also shared a belief that care-giving was natural because it was the ultimate expression of love for a family member. Although often expressed in terms of morality and relationship reciprocity as indicated above, it was done out of love and with the recognition that:

You don't lose by giving, even though you shouldn't expect something [in return].

[FCG12; Int 4]

The value of family-centred care was premised on and tended to reinforce gender- and generational-specific roles and responsibilities. For example, Vietnamese household structures typically follow traditional gender roles, such that domestic and family care responsibilities are performed by women, while the primary bread-winning role is performed by men. The ability and preference to perform gender-specific tasks associated with care were seen as very important within these traditional structures of care. Thus, providing care not only demonstrated a very deep-rooted and unequivocal commitment to the family unit, but helped to fulfil, in part, what it means ‘to be’ Vietnamese, and ‘to be’ a Vietnamese woman. For example:

I am a Vietnamese person, so I have to take care of my mom. You can't just leave her. The doctor has already said that to me. My father works and my husband has to work, right? And therefore because I am the daughter, I have to provide care. A special care because we have to take her to the washroom. Special because we are girls. There is no way a boy go and wipe bottoms or do this and that, or bathe and wash for her. So I have to do it, that's it.

[FCG14; Int 1]

Furthermore, the Vietnamese tend to place a high value on sons within the family hierarchy. This links the expectation that the eldest son will assume responsibility for ageing or ill parents, while the associated household and hands-on personal care will be provided by the daughter-in-law. However, strict adherence to this tradition in Canada was far less rigid:

So the daughter-in-law, according to the Vietnamese tradition, has a greater responsibility towards the husband's family than her own. But the present time, having come to Canada, it seems as though the responsibility is half for the husband's family and half for the wife's family. It's the same.

[FCG01; Int 1]

Thus, arrangements for care were often negotiated among siblings ‘in a practical manner’ [KI05; Int 1], such that daughters, particularly those in this study who were unemployed or who were involved in contingent or precarious employment, were also just as likely to provide care (in fact, as indicated in Table 1, we only recruited one care-giving daughter-in-law to this study). Those female family members who had a reputed, permanent career were less likely to be expected to be a caregiver. This change in expectations was welcome, as some participants expressed sadness around the perceived greater value of males in society. So when traditional care-giving arrangements could not be met by adult sons, there was room for negotiation:

They [my brothers] said that they would take her into the nursing home. I wouldn't allow it, so they said if you don't want to take her [to the nursing home] then you have to care for her. And I said yes and they agreed [to provide her with financial support that enabled her to provide care to her mother at home].

[FCG09; Int 2]

Structures for care-giving also encompassed decision-making processes concerning treatments, settings for care (i.e. home vs. institutional) or funeral arrangements. Such decisions were typically a family affair, although this generally referred to the immediate family only. For the participants in this study, care-giving was perceived as being natural and normal, indicative of the deep-rooted commitment to the family unit; inextricably connected to these perceptions for care were very specific beliefs and values about what care should encompass and how it should be achieved.

### Intentional: whole-person care

Participants shared a belief that health outcomes, including recovery and longevity, are linked not only to medical needs but also to emotional and spiritual well-being. As a consequence, their actions were dedicated towards whole-person care. This care was all encompassing, involving a combination of appropriate medical and alternative treatments, exercise, food/nutrition and emotional and/or spiritual support.

In terms of physical health, care-giving centred on tasks associated with personal care, such as exercising, bathing and administering medications. Cleanliness was paramount [KI05; Int 1]; maintaining this standard was articulated as a source of pride for these caregivers. Furthermore, caregivers nurtured the use of traditional medicine together with western medicine to meet as many patient needs and preferences as possible, as will be elaborated on below.

Food and nutrition were of major symbolic and practical relevance and value to Vietnamese culture in general, and to care in particular. For example, food and ‘being fed’ appear to be more important to Vietnamese people than the acquisition of material items [K107; Int 2]. Food is a major conduit through which expressions of care are transferred and received, as it brings comfort to both those who prepare and those who consume the meals. Traditional foods, such as rice and congee, are more palatable and much easier to digest; however, their nutritional value is not always conducive to their medical conditions. This creates angst for FCGs who struggle with what is ‘right’ in terms of health versus their desire to please the care recipient. As part of their whole-person approach to care, FCGs shared a belief that positive mental health was essential. For many of the care recipients, depression was not only experienced through the ‘loss of self’ from ageing and/or illness, but also through the legacy of loss of family, friends, worldly possessions and their homeland – following the war and subsequent diaspora:

She often remembers things from the past and she would say I lost all my money and they took all my money and *that is why I suffered*. So she lost everything.

[FCG09; Int 3, our emphasis]

Thus, emotional support was not only critical, but very complicated. FCGs described how this typically involved retelling happy stories of the past, avoiding the topic of dying and death, and providing religious or spiritual support through prayer. In addition, this required intentionality by the FCGs to remain calm and patient themselves, both to cope and to prevent care recipients from feeling bad about their condition, behaviour or the extent of care required.

The ways in which physical, emotional and spiritual care are linked to well-being and the intentionality of care-giving are illustrated in the following quotation:

You have to pray. Everything is related to a faithful heart … ‘Let my mom get well and have strength so that she can take her medications so that she can be strong and live with us’ … You pray, but you also have to give her medication, give her food so that it *converges together*. If you only pray and you don't give her the medication or food to eat, how can she be well?

[FCG09; Int 1; our emphasis]

As can be seen, beliefs about appropriate forms of care further shaped FCGs’ values and commitment to the care-giving role. Doing so, however, required a high degree of energy, which was impacted by FCGs’ access to formal and informal support.

### Intensive: standards, struggles and the context of care

Cultural imperatives and individual motivations, combined with a holistic approach to care, necessitated that caregivers invest considerable physical and emotional energy into the care-giving role. Experiences and outcomes of care-giving in this context were thus shaped not only by the magnitude of care but also by the availability and efficacy of formal and informal supports (both real and perceived), as well as caregivers’ willingness to use these support structures.

For example, the perceived need to maintain privacy prevented many FCGs from asking for outside help. This resulted in silent suffering to protect the feelings of the care recipient and manage what is considered a duty towards one's family. FCGs also struggled with care recipients’ preferences to avoid using some services, such as home-care workers. Although this preference is linked to tradition, it is far more complex. As can be seen, the refusal to accept or use services may be part of a deeper issue that can, with communication and creativity, be resolved. Doing so enabled this caregiver to take advantage of respite services:

For example, we are shy about [when] someone showers us … as Vietnamese we're shy letting people shower us. So, at first I bathed him, then I let the personal care worker assisting [sic] him after I started to get ill; he didn't agree, so I'd tell him to leave [on] his underwear….

[FCG08; Int 3]

Vietnamese families value and appreciate the services, and supports that are available in Canada, both within and beyond those related to the healthcare situation specifically. They are, therefore, not likely to complain about what is available, or expect more than they are already offered. In fact, they are more likely to decline something altogether if it is deemed inappropriate, so that the service is not ‘wasted’ and can be given to someone else in need. For example, given that home-care workers are not allowed to administer medications or lift clients on their own, one daughter explained why she ended up refusing home-care services for her bedridden mother:

Home care came for 1 hour two times a week. But they didn't speak Vietnamese, so I would still have to be here. They can't speak [communicate with the care recipient] and they can't lift her, so *I still had to be here to help*. [So] what's the point? Someone else may as well have them [the services].

[FCG17; Int 2, our emphasis]

The above-noted quotation is indicative of the many barriers that caregivers and care recipients experienced in accessing services that led to an increased intensity in care-giving. For example, language barriers (such as the limited or complete inability to read or speak English) and culturally-inappropriate services led to confusion in medical encounters and to insufficient knowledge concerning how to administer medications or access services if language interpreters were not available. For example, one caregiver indicated her desire to better understand the medications prescribed to her mother by the specialist so as to minimise possible side effects and avoid adverse food reactions. Not only did this lack of understanding create unnecessary stress, but making additional arrangements to access this information created extra work, such as through having to make a *further* appointment with her Vietnamese family doctor:

Because they know our language and they can understand me, so they are able to explain things better to me. They are able to understand me more. The specialist – they only prescribe the medication because I don't know the language as much, so they can't explain things to us.

[FCG14; Int 2]

Other strategies used by caregivers to overcome language barriers included scheduling appointments when English-speaking relatives, such as adult-children, could be present during appointments, either in person or over the phone, or having physicians and home-care professionals speak directly with English-speaking relatives over the phone to communicate information. When these strategies failed, caregivers explained how they would have to cancel appointments and other plans because of the communication barriers.

Furthermore, as indicated in Table 1, many of the caregivers in this study were of modest financial means, greatly influencing the amount and types of services and supports – outside those already provided through the public healthcare system – that could be paid for personally. These circumstances created further emotional, physical and financial stress for caregivers, such as through financial hardship because of the purchase, or through extra work because of their inability to pay for what was needed.

Many caregivers experienced a disconnect between available services and preferred services. For example, use of both western and traditional medicines by the Vietnamese is commonplace. Yet, official linkages between these two health and healing systems are lacking, making it difficult to access the type of care that is useful and congruent with their belief systems. This often created stress for caregivers who were worried about how doctors would react, as well as the effects of combining therapies, given that professionals and providers from both traditions tend to practise exclusive of one another, rather than in any integrated manner. In some cases, caregivers withheld the fact that they were using both therapies; others felt that they had to choose:

But my husband's brother said that now my mom-in-law must need to hear the doctors because the doctors give her medicine and something and he didn't want to make trouble with the doctor. He didn't want to take her to the Chinese person to do that [anymore].

[FCG01; Int 1]

And finally, a disconnect was evident in institutional settings, adding to the intensity of care-giving. It materialised not only as a result of communication barriers with healthcare providers but also because of real and perceived understandings concerning the quality of care. For example, caregivers did not believe that care recipients in hospitals and nursing homes would receive the level of care needed, especially compared with what they could provide at home. In addition to quality of care concerns, caregivers were troubled about communication barriers that rendered care recipients lonely, and spoke about how the lack of Vietnamese food negatively impacted overall comfort and well-being.

While motivations and approaches to care, as informed by cultural norms, provide a framework through which care-giving can be defined and structured, they do, together with the availability of culturally-appropriate formal and informal support, significantly impact the intensity of care-giving.

## Discussion and conclusions

Recognising the limitations of the study, with respect to the variability around the care-giving experience (i.e. degree of service provision and amount of informal support available), the issues of information retrieval for bereaved caregivers and the probability of error in given translation and transcription, to our knowledge, this is first ‘world’ view of the experience of family care-giving from the perspectives of Vietnamese FCGs living in Canada. The findings illustrate, as with other cultural traditions, the ‘naturalness’ of care-giving and how these experiences are filtered through FCGs’ social and cultural identities. However, the ability of Vietnamese FCGs to provide care in accordance with these known traditions is at risk due to the changing social and cultural milieu in which they find themselves in Canada. The ability to sustain the traditional culture of care has changed immensely, as Vietnamese families adapt and assimilate into Canadian society. For example, expectations to acquire an education, the need for both spouses to work to provide the necessities of life, combined with the desire by younger or Canadian-born children to have greater autonomy, have reduced the willingness and availability of families to provide care, as is still expected in Vietnam. Older FCGs may not experience the loss of tradition to the same extent as younger generations who may be under more pressure to seek outside help to manage their responsibilities. Such circumstances put adult-children caregivers in conflict with the expectations of aged and ill parents and with what is possible within Canadian society (Spitzer *et al.* 2003, Lai 2007). Although informal family care-giving is highly valued within Vietnamese culture, as with many Asian cultures, it is devalued within the context of our economic system. For example, social and economic pressures to earn an income make it difficult to provide the type and level of care Vietnamese FCGs perceive as necessary and appropriate. The devaluing of FCGs is a common trend within the context of healthcare restructuring; it remains a fundamental flaw of the shift to community-based care because of the reliance on FCGs for success.

This work illustrates how the experience of care-giving within a Vietnamese cultural tradition can impact FCGs’ overall mental and physical health. The intensity of care, as a function of cultural traditions and the limited receipt of formal services, illustrates that there is currently a high degree of burden among these caregivers, and that this ‘group’ is at risk, in general, for increased burden, resulting in negative impacts on the physical, emotional, social and financial well-being. As with other cultures, women are at particular risk, given the gendered expectations to care and limited social networks of support. Although these caregivers illustrated a high degree of resilience and independence, they are extremely vulnerable to burden and exploitation. In keeping with the PHP model, this research confirms the need for culturally-appropriate health and social services and supports for Vietnamese families offered across multiple scales, including the individual FCG, the family, the community, and the health and social sectors/systems more broadly. Such culturally-appropriate services are characterised as language-accessible; family-oriented (rather than patient-centred); respectful of privacy and modesty; and tolerant of multiple healthcare approaches (i.e. both western and traditional medicine), as well as specific food preferences. It illustrates that Vietnamese FCGs not only value, but are also likely to use healthcare and social services, if they are language-accessible; affordable; useful; and demonstrate respect for their values as individuals.
